# Supplementary material for: Identification and Quantification of Coumarins by UHPLC-MS in Arabidopsis thaliana Natural Populations
Source: Molecules. 2021 Mar 23;26(6):1804. doi: 10.3390/molecules26061804 (PMC8005220; doi:10.3390/molecules26061804)
Supplement: Supplementary file 1 [file molecules-26-01804-s001.pdf]

Table S1. The mean values (scopoletin and scopolin concentrations) and standard deviations (±SD) that were used in making heat maps shown in Figure 2.

	Scopoletin concentration [μM]				Scopolin concentration [μM]			
**Accession**	**roots, H-**	**roots, H+**	**leaves, H-**	**leaves, H+**	**roots, H-**	**roots, H+**	**leaves, H-**	**leaves, H+**
**An-1**	4.91 ± 4.12	15.19 ± 11.34	0.10 ± 0.12	0.45 ± 0.24	14.81 ± 4.05	0.13 ± 0.12	0.68 ± 0.41	0.05 ± 0.08
**Bay-0**	41.93 ± 6.58	151.90 ± 30.12	0.29 ± 0.18	1.99 ± 1.46	38.95 ± 10.69	07.15 ± 6.36	3.81 ± 1.22	0.88 ± 1.53
**Br-0**	35.01 ± 9.48	139.27 ± 49.38	0.31 ± 0.09	2.61 ± 0.27	67.26 ± 23.82	1.22 ± 0.08	1.65 ± 0.08	0
**C24**	4.92 ± 2.67	16.63 ± 10.42	0.20 ± 0.11	0.83 ± 0.55	41.36 ± 24.8	0.43 ± 0.19	0.85 ± 0.59	0
**Can-0**	3.14 ± 1.89	64.03 ± 84.91	0.14 ± 0.14	0.37 ± 0.12	34.63 ± 4.85	0.56 ± 0.05	2.23 ± 1.24	0.19 ± 0.33
**Col-0**	11.79 ± 3.16	29.60 ± 11.41	0.31 ± 0.41	1.16 ± 0.99	11.47 ± 3.41	0.46 ± 0.07	1.48 ± 0.67	0
**Cvi-1**	4.58 ± 0.62	11.54 ± 7.57	0.19 ± 0.10	0.61 ± 0.27	35.41 ± 32.83	0.35 ± 0.16	2.15 ± 0.8	0
**Eil-0**	8.03 ± 4.90	21.55 ± 14.72	0.15 ± 0.03	0.53 ± 0.23	28.24 ± 11.10	1.12 ± 0.2	2.19 ± 1.16	0.02 ± 0
**Eri-0**	12.86 ± 3.96	23.84 ± 13.33	0.13 ± 0.09	0.68 ± 0.48	20.55 ± 8.45	2.04 ± 0.3	2.24 ± 0.93	0.03 ± 0.04
**Est-1**	14.85 ± 1.63	36.79 ± 2.44	0.42 ± 0.19	2.01 ± 0.86	32.22 ±12.66	0.44 ± 0.2	6.48 ± 3.7	0.07 ± 0.09
**Fei-0**	12.82 ± 8.73	52.02 ± 15.32	0.09 ± 0.06	0.73 ± 0.37	62.36 ± 28.64	0.52 ± 0.08	2.77 ± 1.53	0.08 ± 0.13
**Fuk-1**	13.06 ± 7.87	44.36 ± 47.37	0.13 ± 0.01	0.62 ± 0.11	29.23 ± 27.52	8.09 ± 7.02	1.58 ± 0.62	0.05 ± 0.09
**Ga-0**	3.40 ± 1.11	19.67 ± 12.65	0.03 ± 0.01	0.55 ± 0.16	53.08 ± 7.05	1.29 ± 0.25	3.03 ± 0.58	0.12 ± 0.25
**Hog**	7.47 ± 7.02	20.31 ± 20.17	0.10 ± 0.06	0.34 ± 0.12	16.9 ± 14.41	0.85 ± 0.52	1.03 ± 0.46	0
**Kas-2**	4.95 ± 0.33	22.71 ± 7.70	0.04 ± 0.01	0.39 ± 0.21	21.39 ± 17.39	0.59 ± 0.26	1.54 ± 0.63	0.24 ± 0.41
**Kondara**	42.79 ± 9.84	81.30 ± 25.39	0.26 ± 0.27	1.05 ± 0.29	25.10 ± 16.58	0.86 ± 0.71	0.80 ± 0.14	0.04 ± 0.08
**Kyo-0**	5.20 ± 1.63	26.87 ± 18.45	0.04 ± 0.04	0.82 ± 0.53	33.97 ± 12.23	0.77 ± 0.26	3.50 ± 2.50	0
**Leb 3/4**	17.93 ± 5.89	34.91 ± 19.59	0.19 ± 0.11	0.72 ± 0.36	41.77 ± 27.88	0.84 ± 0.19	2.26 ± 1.41	0
**Ler-1**	3.88 ± 1.52	8.89 ± 2.94	0.04 ± 0.07	0.27 ± 0.05	2.94 ± 0.66	0.23 ± 0.07	0.69 ± 0.07	0.16 ± 0.27
**No-0**	2.61 ± 1.17	8.35 ± 8.20	0.19 ± 0.14	1.10 ± 0.92	10.26 ± 6.89	1.09 ± 0.16	3.41 ± 1.92	0.06 ± 0.11
**Ri-0**	11.42 ± 4.05	44.55 ± 21.08	0.13 ± 0.06	0.48 ± 0.49	41.47 ± 25.01	5.15 ± 0.93	2.06 ± 1.03	0
**Sf-2**	5.12 ± 3.51	11.29 ± 8.85	0.05 ± 0.03	0.22 ± 0.14	10.67 ± 8.42	0.42 ± 0.15	0.99 ± 0.29	0
**Sha-1**	15.46 ± 0.67	36.68 ± 2.00	0.15 ± 0.11	0.73 ± 0.56	31.31 ± 6.11	3.16 ± 0.89	2.32 ± 1.99	0.15 ± 0.26
**Sorbo**	15.17 ± 4.57	26.08 ± 16.16	0.24 ± 0.26	1.35 ± 0.83	9.12 ± 6.08	0.99 ± 0.27	5.01 ± 3.04	0.69 ± 0.85
**Ts-5**	4.75 ± 0.38	16.47 ± 14.26	0.08 ± 0.07	0.92 ± 0.55	7.89 ± 4.64	0.64 ± 0.13	0.92 ± 0.61	0
**Tsu-1**	2.71 ± 0.78	10.32 ± 1.96	0.03 ± 0.02	0.14 ± 0.04	12.53 ± 6.06	0.87 ± 0.33	0.35 ± 0.09	0.01 ± 0.01
**Van-0**	4.01 ± 3.01	8.02 ± 11.19	0.10 ± 0.09	0.77 ± 0.93	7.98 ± 6.97	0.24 ± 0.09	2.30 ± 1.33	0.13 ± 0.12
**Ws-0**	9.68 ± 5.92	19.16 ± 18.60	0.16 ± 0.12	0.54 ± 0.12	30.49 ± 27.29	0.58 ± 0.15	2.58 ± 0.36	0.26 ± 0.24

Table S2. The mean values (umbelliferone and skimmin concentrations) and standard deviations (±SD) that were used in making heat maps shown in Figure 3.

	Umbelliferone concentration [μM]				Skimmin concentration [μM]			
**Accession**	**roots, H-**	**roots, H+**	**leaves, H-**	**leaves, H+**	**roots, H-**	**roots, H+**	**leaves, H-**	**leaves, H+**
**An-1**	0.02 ± 0.03	0.23 ± 0.15	0	0	3.53 ± 2.92	0.23 ± 0.34	0.30 ± 0.03	0
**Bay-0**	0.16 ± 0.08	1.64 ± 0.14	0	0	7.32 ± 1.51	5.40 ± 2.98	0	0
**Br-0**	0.06 ± 0.02	1.05 ± 1.01	0	0	3.19 ± 1.58	0.59 ± 0.17	0	0
**C24**	0.06 ± 0.11	0.97 ± 0.59	0	0	15.25 ± 9.47	3.48 ± 4.57	0.30 ± 0.04	0
**Can-0**	0	0.30 ± 0.36	0	0	2.59 ± 0.61	0.76 ± 0.57	0.07 ± 0.05	0
**Col-0**	0.15 ± 0.10	1.06 ± 0.17	0	0	8.40 ± 5.60	0.43 ± 0.41	0.29 ± 0.24	0.07 ± 0.08
**Cvi-1**	0	0.07 ± 0.05	0	0	2.70 ± 2.16	0.13 ± 0.11	0	0
**Eil-0**	0	0.09 ± 0.04	0	0	6.38 ± 2.34	0.60 ± 0.48	0	0
**Eri-0**	0.14 ± 0.06	0.42 ± 0.27	0	0	9.76 ± 2.83	0.68 ± 0.69	0.34 ± 0.13	0
**Est-1**	0.07 ± 0.09	1.09 ± 0.67	0	0	12.27 ± 0.26	1.64 ± 0.55	0	0
**Fei-0**	0.05 ± 0.04	0.31 ± 0.08	0	0	3.42 ± 2.05	0.37 ± 0.41	0	0
**Fuk-1**	0.10 ± 0.09	0.72 ± 0.60	0	0	1.34 ± 0.42	2.81 ± 3.43	0.10 ± 0.05	0
**Ga-0**	0	0.20 ± 0.16	0	0	19.80 ± 3.01	2.00 ± 1.49	0.13 ± 0.11	0
**Hog**	0	0.03 ± 0.04	0	0	5.87 ± 1.67	1.43 ± 0.87	0	0
**Kas-2**	0	0.63 ± 0.18	0	0	2.62 ± 0.61	0.09 ± 0.05	0	0
**Kondara**	0.02 ± 0.04	0.50 ± 0.38	0	0	5.11 ± 1.26	0.36 ± 0.28	0.19 ± 0.10	0.01 ± 0.02
**Kyo-0**	0	0.40 ± 0.27	0	0	11.60 ± 3.00	1.14 ± 0.08	0	0
**Leb 3/4**	0.11 ± 0.02	0.72 ± 0.38	0	0	4.25 ± 2.36	0.20 ± 0.13	0	0
**Ler-1**	0.03 ± 0.02	0.34 ± 0.11	0	0	2.21 ± 0.57	0.48 ± 0.07	0	0
**No-0**	0	0.03 ± 0.05	0	0	8.88 ± 6.51	2.49 ± 2.48	0	0
**Ri-0**	0.14 ± 0.06	1.41 ± 0.68	0	0	13.25 ± 4.35	18.76 ± 16.26	0	0
**Sf-2**	0	0.29 ± 0.11	0	0	1.96 ± 1.58	0.30 ± 0.27	0	0
**Sha-1**	0.07 ± 0.03	0.63 ± 0.18	0	0	8.01 ± 1.30	0.93 ± 0.80	0	0
**Sorbo**	0.24 ± 0.09	0.78 ± 0.35	0	0	1.52 ± 0.44	0.04 ± 0.05	0	0
**Ts-5**	0	0.38 ± 0.31	0	0	0.69 ± 0.05	0.23 ± 0.11	0	0
**Tsu-1**	0	0.29 ± 0.07	0	0	5.82 ± 3.00	0.51 ± 0.35	0.04 ± 0.06	0
**Van-0**	0.02 ± 0.04	0.63 ± 0.56	0	0	4.38 ± 0.91	0.32 ±0.20	0.25 ± 0.13	0.04 ± 0.06
**Ws-0**	0.11 ± 0.04	0.45 ± 0.31	0	0	14.04 ± 3.26	1.42 ± 0.24	0.17 ± 0.08	0.03 ± 0.06

Table S3. The mean values (esculetin and esculin concentrations) and standard deviations (±SD) that were used in making heat maps shown in Figure 4.

	Esculetin concentration [μM]				Esculin concentration [μM]			
**Accession**	**roots, H-**	**roots, H+**	**leaves, H-**	**leaves, H+**	**roots, H-**	**roots, H+**	**leaves, H-**	**leaves, H+**
**An-1**	0	0	0.04 ± 0.07	0	0	0	0.02 ± 0.01	0
**Bay-0**	0.11 ± 0.18	0.26 ± 0.26	0	0	0	0	0	0
**Br-0**	0.07 ± 0.12	0	0.14 ± 0.03	0	0	0	0	0
**C24**	0	0	0.16 ± 0.03	0	0	0	0.01 ± 0.01	0
**Can-0**	0.01 ± 0.02	0.29 ± 0.51	0	0	0	0	0.09 ± 0.15	0.15 ± 0.13
**Col-0**	0	0.20 ± 0.14	0.02 ± 0.03	0	0	0.01 ± 0.01	0.15 ± 0.21	0.08 ± 0.15
**Cvi-1**	0	0	0	0	0	0	0	0
**Eil-0**	0	0	0	0	0	0	0	0
**Eri-0**	0	0	0	0	0	0	0	0.07 ± 0.12
**Est-1**	0	0	0	0	0	0	0.12 ± 0.02	0
**Fei-0**	0	0	0	0	0	0	0	0
**Fuk-1**	0	0	0	0	0	0	0	0
**Ga-0**	0	0	0	0	0	0	0	0
**Hog**	0	0	0	0	0	0	0.01 ± 0.01	0
**Kas-2**	0	0	0	0	0	0	0.03 ± 0.05	0
**Kondara**	0	0	0	0	0	0	0	0
**Kyo-0**	0	0	0	0	0	0	0	0
**Leb 3/4**	0	0	0	0	0	0	0	0
**Ler-1**	0	0	0	0	0	0	0.01 ± 0.01	0.03 ± 0.06
**No-0**	0	0	0.02 ± 0.03	0	0	0	0.02 ± 0.04	0
**Ri-0**	0	0.20 ± 0.06	0.01 ± 0.02	0	0	0	0.04 ± 0.07	0
**Sf-2**	0	0	0	0	0	0	0	0
**Sha-1**	0	0	0	0	0	0	0	0
**Sorbo**	0	0	0	0	0	0	0	0
**Ts-5**	0	0	0	0	0	0	0	0.01 ± 0.02
**Tsu-1**	0	0.08 ± 0.08	0	0	0	0	0	0
**Van-0**	0	0	0	0	0	0	0	0.06 ± 0.08
**Ws-0**	0	0	0.02 ± 0.02	0.01 ± 0.02	0	0	0.01 ± 0.02	0.36 ± 0.32

Table S4. Coumarin concentrations in root samples before hydrolysis and four geographic and climatic factors used in principle component analysis (PCA). Average winter temperatures – taken from November to April; average summer temperatures – taken from May to October. Altitude and annual precipitation data are taken for the closest place where the given accession originates. Data sources [1] Temperature and precipitation (Temperature and precipitation - https://www.climatestotravel.com/ (US: https://www.usclimatedata.com/climate/columbia/north-carolina/united-states/usnc0145), [2] Altitude (https://www.freemaptools.com/elevation-finder.htm), [3] Annual precipitation (https://weather-and-climate.com/).

Altitude max	AV winter min temp [°C]	AV summer max temp [°C]	AV annual precipitation [mm]	Umbelliferone	Skimmin	Scopoletin	Scopolin	Continent	Accession
100	2.5	20	850	0.231	0.014	15.186	0.006	Europe	An-1
400	-0.7	20.7	640	1.644	0.323	151.905	0.336	Europe	Bay-0
300	-0.8	21.3	493	1.05	0.036	139.274	0.057	Europe	Br-0
30	6.7	25.3	905	0.972	0.208	16.627	0.02	Europe	C24
1260	16	26.2	146	0.3	0.046	64.031	0.026	Africa	Can-0
100	3.85	27.9	1265	1.056	0.025	29.604	0.021	North America	Col-0
1200	19.8	26	97	0.067	0.008	11.538	0.016	Africa	Cvi-1
200	0.3	20.5	535	0.086	0.036	21.551	0.052	Europe	Eil-0
119	-1.5	17.8	660	0.418	0.041	23.835	0.096	Europe	Eri-1
200	-4.2	16.5	695	1.088	0.098	36.793	0.021	Europe	Est-1
140	7	22.7	1255	0.311	0.022	52.02	0.025	Europe	Fei-0
18	5.2	27.7	1540	0.721	0.475	44.36	0.379	Asia	Fuk-1
200	1.3	21.5	620	0.199	0.12	19.67	0.06	Europe	Ga-0
1887	2.3	30.5	610	0.078	0.085	20.311	0.04	Asia	Hog
1580	-1.8	27	215	0.625	0.005	22.713	0.028	Asia	Kas-2
916	2.3	30.5	610	0.499	0.022	81.3	0.04	Asia	Kondara
98	4.3	28.3	1490	0.4	0.068	26.866	0.036	Asia	Kyo-1
301	-14.6	21.2	264	0.718	0.012	34.909	0.039	Asia	Leb 3/4
614	0.3	20.5	535	0.335	0.028	8.892	0.011	Europe	Ler-1
300	0.33	20.3	665	0.089	0.149	8.353	0.051	Europe	No-0
100	2.7	19	1190	1.41	0.000	44.551	0.648	North America	Ri-0
100	6.8	25.2	620	0.288	0.018	11.291	0.02	Europe	Sf-2
3400	-4.2	25.3	233	0.627	0.056	36.684	0.148	Asia	Sha-1
646	-4.3	25.4	234	0.781	0.002	26.081	0.046	Asia	Sorbo
100	6.8	25.2	620	0.382	0.014	16.469	0.03	Europe	Ts-5
100	4.5	27.8	1535	0	0.348	2.711	0.588	Asia	Tsu-1
100	2.7	19	1190	0.628	0.019	8.022	0.011	North America	Van-0
200	-3.5	19.2	675	0.446	0.085	19.157	0.027	Europe	Ws-0
